# Clinical Outcomes After Acute Coronary Syndromes or Revascularization Among People Living With HIV: A Systematic Review and Meta-Analysis

**DOI:** 10.1001/jamanetworkopen.2024.11159

**Published:** 2024-05-14

**Authors:** Mohammed Haji, Michael Capilupi, Michael Kwok, Nouran Ibrahim, Gerald S. Bloomfield, Christopher T. Longenecker, Maria C. Rodriguez-Barradas, Chester N. Ashong, Eric Jutkowitz, Tracey H. Taveira, Michelle Richard, Jennifer L. Sullivan, James L. Rudolph, Wen-Chih Wu, Sebhat Erqou

**Affiliations:** 1 Department of Medicine, Alpert Medical School of Brown University, Providence, Rhode Island; 2 Department of Medicine, Washington University School of Medicine in St Louis, St Louis, Missouri; 3 Department of Medicine, Duke Global Health Institute and Duke Clinical Research Institute, Duke University, Durham, North Carolina; 4 Global Health Institute, University of Washington, Seattle; 5 Infectious Disease Section, Michael E. DeBakey VA Medical Center, Houston, Texas; 6 Department of Medicine, Baylor College of Medicine, Houston, Texas; 7 Pharmacy Service, Michael E. DeBakey VA Medical Center, Houston, Texas; 8 Center of Innovation, Providence VA Medical Center, Providence, Rhode Island; 9 Evidence Synthesis Program Center, Providence VA Health Care System, Providence, Rhode Island; 10 Department of Medicine, Providence VA Medical Center, Providence, Rhode Island; 11 Department of Pharmacy, University of Rhode Island, Providence; 12 Department of Health Services, Policy and Practice, Brown University, Providence, Rhode Island

## Abstract

**Importance:**

Clinical outcomes after acute coronary syndromes (ACS) or percutaneous coronary interventions (PCIs) in people living with HIV have not been characterized in sufficient detail, and extant data have not been synthesized adequately.

**Objective:**

To better characterize clinical outcomes and postdischarge treatment of patients living with HIV after ACS or PCIs compared with patients in an HIV-negative control group.

**Data Sources:**

Ovid MEDLINE, Embase, and Web of Science were searched for all available longitudinal studies of patients living with HIV after ACS or PCIs from inception until August 2023.

**Study Selection:**

Included studies met the following criteria: patients living with HIV and HIV-negative comparator group included, patients presenting with ACS or undergoing PCI included, and longitudinal follow-up data collected after the initial event.

**Data Extraction and Synthesis:**

Data extraction was performed following the Preferred Reporting Items for Systematic Reviews and Meta-Analysis (PRISMA) statement. Clinical outcome data were pooled using a random-effects model meta-analysis.

**Main Outcome and Measures:**

The following clinical outcomes were studied: all-cause mortality, major adverse cardiovascular events, cardiovascular death, recurrent ACS, stroke, new heart failure, total lesion revascularization, and total vessel revascularization. The maximally adjusted relative risk (RR) of clinical outcomes on follow-up comparing patients living with HIV with patients in control groups was taken as the main outcome measure.

**Results:**

A total of 15 studies including 9499 patients living with HIV (pooled proportion [range], 76.4% [64.3%-100%] male; pooled mean [range] age, 56.2 [47.0-63.0] years) and 1 531 117 patients without HIV in a control group (pooled proportion [range], 61.7% [59.7%-100%] male; pooled mean [range] age, 67.7 [42.0-69.4] years) were included; both populations were predominantly male, but patients living with HIV were younger by approximately 11 years. Patients living with HIV were also significantly more likely to be current smokers (pooled proportion [range], 59.1% [24.0%-75.0%] smokers vs 42.8% [26.0%-64.1%] smokers) and engage in illicit drug use (pooled proportion [range], 31.2% [2.0%-33.7%] drug use vs 6.8% [0%-11.5%] drug use) and had higher triglyceride (pooled mean [range], 233 [167-268] vs 171 [148-220] mg/dL) and lower high-density lipoprotein-cholesterol (pooled mean [range], 40 [26-43] vs 46 [29-46] mg/dL) levels. Populations with and without HIV were followed up for a pooled mean (range) of 16.2 (3.0-60.8) months and 11.9 (3.0-60.8) months, respectively. On postdischarge follow-up, patients living with HIV had lower prevalence of statin (pooled proportion [range], 53.3% [45.8%-96.1%] vs 59.9% [58.4%-99.0%]) and β-blocker (pooled proportion [range], 54.0% [51.3%-90.0%] vs 60.6% [59.6%-93.6%]) prescriptions compared with those in the control group, but these differences were not statistically significant. There was a significantly increased risk among patients living with HIV vs those without HIV for all-cause mortality (RR, 1.64; 95% CI, 1.32-2.04), major adverse cardiovascular events (RR, 1.11; 95% CI, 1.01-1.22), recurrent ACS (RR, 1.83; 95% CI, 1.12-2.97), and admissions for new heart failure (RR, 3.39; 95% CI, 1.73-6.62).

**Conclusions and Relevance:**

These findings suggest the need for attention toward secondary prevention strategies to address poor outcomes of cardiovascular disease among patients living with HIV.

## Key Points

**Question:**

What are the postdischarge outcomes for patients living with HIV after acute coronary syndromes or coronary revascularization?

**Findings:**

In this systematic review and meta-analysis of 15 studies involving 9499 patients living with HIV and 1 531 117 patients without HIV, patients living with HIV had a higher risk of all-cause mortality, major adverse cardiovascular events, recurrent acute coronary syndromes, and admission for heart failure after the index event, despite being approximately 11 years younger at the time of the event. Patients living with HIV were more likely to be current smokers and engage in illicit drug use and had higher triglyceride and lower high-density lipoprotein cholesterol levels than those without HIV.

**Meaning:**

This analysis highlights the need for attention toward secondary prevention strategies to address poor outcomes of cardiovascular disease among patients living with HIV.

## Teaser

This systematic review and meta-analysis investigates clinical outcomes and postdischarge treatment after acute coronary syndromes or revascularization among people living with HIV.

## Introduction

The widespread use of effective antiretroviral therapies (ARTs) has led to increased survivorship among people living with HIV. Therefore, people living with HIV are experiencing an increased prevalence of age-related disease, such as cardiovascular disease (CVD).^1,2^ The increase in CVD in this population has been attributed to multiple factors, including increasing age, the increase in burden of traditional CVD factors and psychosocial risk factors, the long-term metabolic effects of ART, and the low-grade immune activation of chronic HIV.^1,3,4,5,6,7,8^

Epidemiological studies have shown that compared with populations without HIV, people living with HIV have a higher risk of coronary artery disease, acute coronary syndromes (ACS), and heart failure, with onset at younger ages.^4,9,10,11,12^ Given this earlier emergence of CVD among people living with HIV, there has been significant attention and evidence generated for primary prevention strategies involving statins.^13,14^ In conjunction with these studies, characterization of longitudinal CVD outcomes is important to identify strategies for secondary prevention and further improve survivorship among people living with HIV. Studies on clinical outcomes after ACS and percutaneous coronary interventions (PCIs) among patients living with HIV have shown higher rates of recurrent coronary disease and mortality compared with patients in HIV-negative control groups.^11,15,16,17^ However, this association has not been characterized in sufficient detail in current literature, and extant data have not been adequately synthesized. We conducted a systematic review and meta-analysis of longitudinal studies of patients living with HIV after ACS or PCIs to better characterize clinical outcomes and postdischarge treatment compared with patients in HIV-negative control groups.

## Methods

We report this systematic review and meta-analysis according to the Preferred Reporting Items for Systematic Reviews and Meta-analyses (PRISMA) reporting guideline. This study was not preregistered. Please see the eMethods in Supplement 1 for a detailed description of methods used in this meta-analysis, as recommended by the International Committee of Medical Journal Editors.

### Search and Extraction

We searched Ovid MEDLINE, Embase, and Web of Science for all available articles from inception to August 2023 for the key terms *coronary artery disease*, *myocardial infarction*, *non-fatal myocardial infarction*, *acute coronary syndrome*, *revascularization*, *percutaneous coronary intervention*, and *secondary prevention*. We also reviewed references of relevant articles.

Articles were screened by 2 reviewers (M.H. and M.C.) by title and abstract and later by full text. We included studies if they fulfilled the following criteria: patients living with HIV and a comparator group of patients without HIV (control group) included, patients with obstructive coronary artery disease presenting with ACS or undergoing revascularization through PCI included, and longitudinal follow-up data on clinical outcomes after initial event collected. We initially also searched for studies that discussed outcomes after stroke and peripheral artery disease.

We extracted the following data where available using standardized forms: study characteristics, baseline demographics (ie, age, sex, and race and ethnicity) and other characteristics (ie, underlying comorbidities, revascularization strategies, and postdischarge medications) of HIV-positive and HIV-negative control populations, HIV-specific characteristics (use of ART, CD4 count, and viral load), number of events by group and hazard ratios (HRs) of clinical outcomes (ie, all-cause mortality, major adverse cardiovascular events [MACE], cardiovascular death, recurrent ACS, stroke, total lesion revascularization, total vessel revascularization, and admission for heart failure). We extracted maximally adjusted HRs where available, as well as unadjusted (crude) or minimally adjusted HRs for clinical outcomes. We captured data on race and ethnicity to help assess the full scope of diversity among patients living with HIV and how applicable our data may be within the global population of people living with HIV. Race and ethnicity were self-reported in the study by Shitole et al.^18^ In the other studies reporting this information, data were obtained from review of medical records, including electronic health records. Reported race and ethnicity categories included African American, American Indian, Asian, Hispanic, Pacific Islander, White, and other. We primarily report aggregated data for Black, White, and Hispanic populations only given that there were limited data available on other races and ethnicities.

### Statistical Analysis

We combined summary study characteristics (eg, mean age, percentage male and female, percentage Black and White, and percentage Hispanic) across studies using study sizes as analytical weights to provide estimates of pooled means or percentages. The δ and *P* values comparing summary study-level characteristics (means or prevalences pooled across studies) between HIV-positive and HIV-negative groups were calculated from a linear regression model of each variable on HIV status weighted by the number of participants for each study (ie, a fixed-effects meta-regression). When HRs were not reported, we calculated crude risk ratios from the number of events in each group. In 2 studies,^15,19^ data were reported as odds ratios. We pooled HRs of clinical outcomes across studies using a random-effects model meta-analysis, estimating between-study heterogeneity using the DerSimonian-Laird method.^20^ As a sensitivity analysis, we also estimated between-study heterogeneity using the residual maximum likelihood method and calculated variances (*P* values and CIs) of pooled relative risk (RR) estimates using modifications proposed by Knapp and Hartung.^21^ For the purpose of the meta-analysis, we considered odds ratios, risk ratios, and HRs as equivalent measures of RR.

We assessed between-study heterogeneity using the Cochran *Q* statistic and *I*^2^ statistic, which estimates the percentage of total variation across studies due to true between-study difference rather than chance.^22,23^ We did not explore heterogeneity further owing to the limited numbers of studies available for most comparisons.

The quality of included studies was assessed using the Newcastle-Ottawa Scale for cohort studies.^24^ We visually inspected funnel plots to assess the risk of publication bias. We also performed the Egger test for small study bias, although this was limited by the small number of studies that were generally available for investigated outcomes. Where there were *P* values trending toward small study bias, we performed trim and fill analyses to help assess the impact of the bias on pooled estimates (even if Egger test *P* values did not reach statistical significance). A 2-sided *P* value less than .05 was considered statistically significant. For the meta-analysis of RRs, we report point estimates and 95% CIs. All analyses were performed using Stata software statistical software version 15 (StataCorp).

## Results

An initial search yielded 3263 studies, which were screened using titles, abstracts, and full texts. Studies reviewing patient outcomes after diagnoses and interventions of peripheral artery disease and stroke were limited, reporting mainly in-hospital outcomes, short-term follow-up, or results without non-HIV comparator groups, and were not further considered in this meta-analysis. We identified 15 studies^11,15,16,18,25,26,27,28,29,30,31,32,33,34,35^ of post-ACS or revascularization outcomes from 2003 to 2023 that met inclusion criteria (eFigure 1 in Supplement 1). Of identified studies, 2 were abstracts.^30,31^ All were retrospective cohort studies except for 3 prospective studies (Table 1).^11,26,30^

**Table 1.** Study Characteristics, Patient Characteristics, and Outcomes

Characteristic	Study														
	Matetzky et al,^26^ 2003	Hsue et al,^34^ 2004	Ren et al,^27^ 2009	Lorgis et al,^15^ 2013	Carballo et al,^16^ 2015	Badr et al,^32^ 2015	Jeon et al,^25^ 2017	Mandal et al,^31^ 2017	Cua et al,^30^ 2014	Marcus et al,^29^ 2019	Boccara et al,^11^ 2020	Shitole et al,^18^ 2020	Postigo et al,^28^ 2020	Parks et al,^33^ 2021	Parikh et al^35^ 2023
Study date	1998-2000	1993-2003	2000-2007	2005-2009	2005-2011	2003-2011	2002-2014	2003-2016	2002-2010	1996-2010	2003-2006	2008-2014	2000-2018	2014-2016	2009-2019
Study design	PC	COS	RC	RC	RC	COS	RC	COS	PC	RC	PC	RC	RC	COS	RC
Population source	Cedars-Sinai Medical Center, Los Angeles	San Francisco General Hospital	California Pacific Medical Center	PMSI database in France	Swiss HIV Cohort Study	MedStar Washington	Ontario HIV databases	Rural Kolkata, India	Veterans Aging Cohort	Kaiser Permanente	23 CCUs in France	Montefiore Hospital, NY	Gregorio Maranon Hospital, Madrid, Spain	Symphony Health data warehouse	VA Healthcare System
Patients, No.															
Control	48	68	97	1216	5328	112	259 475	32	1564	86 321	195	1152	184	1 118 514	56 811
HIV	24	68	97	608	133	112	345	32	479	226	103	22	92	6612	546
Age, mean, y															
Control	48	61	54	50	64	58	69.4	42.0	NA	67	50	60	51.3	67.4	67.1
HIV	47.0	50	53	50	51	58	54.4	49	NA	54	48	50	51.3	57.4	63.0
Sex,%															
Male															
Control	87.5	61.7	100	88.6	72.2	64.3	61.7	87.5	NA	63	94.3	66.7	92.4	59.7	98.1
HIV	87.5	89.7	100	88.6	85.0	64.3	87.0	93.8	NA	94	93.2	72.7	92.4	71.2	98.9
Female															
Control	12.5	38.2	0	11.4	27.8	35.7	38.3	12.5	NA	37	5.7	33.3	7.6	40.3	1.9
HIV	12.5	10.3	0	11.4	15.0	35.7	13.0	6.2	NA	6	6.8	27.3	7.6	28.8	1.1
Race and ethnicity, %															
Black															
Control	NA	NA	NA	NA	NA	21.4	NA	NA	NA	6.5	NA	20.3	NA	2.5	14.0
HIV	NA	NA	NA	NA	NA	62.5	NA	NA	NA	15	NA	22.7	NA	7.0	34.8
Hispanic															
Control	NA	NA	NA	NA	NA	NA	NA	NA	NA	0.7	NA	36.3	NA	3.8	NA
HIV	NA	NA	NA	NA	NA	NA	NA	NA	NA	0.4	NA	54.6	NA	8.2	NA
White															
Control	NA	NA	NA	NA	NA	NA	NA	NA	NA	68	NA	23.1	NA	14.3	83.7
HIV	NA	NA	NA	NA	NA	NA	NA	NA	NA	64	NA	9.1	NA	7.2	63.4
Outcomes, No.															
MACE															
Control	NA	NA	29	NA	1078	19	NA	1	451	NA	39	425	30	NA	9910
HIV	2	NA	32	NA	20	28	NA	3	143	NA	22	11	17	NA	121
Death															
Control	NA	NA	2	NA	135	12	NA	NA	73	16 401	NA	185	7	114 933	4373
HIV	NA	NA	3	NA	5	17	NA	NA	35	35	NA	3	6	724	57

Abbreviations: CCUs, coronary care unit; COS, comparative observational study; MACE, major adverse cardiac events; NA, not available; PC, prospective cohort; PMSI, Programme de Médicalisation des Systèm’s d'Information; RC, retrospective cohort; VA, Veterans Affairs.

Details of patient characteristics and outcomes by study are presented in Table 1 and eTable 1 in Supplement 1. A total of 9499 patients living with HIV (pooled proportion [range], 76.4% [64.3%-100%] male; pooled mean [range] age, 56.2 [47.0-63.0] years; pooled proportion [range], 10.1% [95% CI, 7.0%-62.5%] Black; 8.1% [95% CI, 0.4%-54.6%] Hispanic, and 13.1% [95% CI, 7.2%-64.0%] White) and 1 531 117 patients in control groups without HIV (pooled proportion [range], 61.7% [59.7%-100%] male; pooled mean [range] age, 67.7 [42.0-69.4] years; pooled proportion [range], 3.3% [95% CI, 2.5%-21.4%] Black, 3.6% [95% CI, 0.7%-36.3%] Hispanic, and 21.1% [95% CI, 14.3%-68.0%] White) who experienced ACS or underwent coronary revascularization were included in the meta-analysis. Summary baseline characteristics of study participants and comparisons of patients living with HIV with patients in control groups are presented in Table 2 and eTable 2 in Supplement 1. The mean age of patients living with HIV was 11.1 years (95% CI, 6.2-16.0 years) less than that of patients in HIV-negative control groups (*P* < .001). HIV-positive and control populations were similarly male dominant. Patients living with HIV were statistically significantly more likely to be current smokers (pooled proportion [range], 59.1% [24.0%-75.0%] smokers vs 42.8% [26.0%-64.1%] smokers; *P* < .001) and engage in illicit drug use (pooled proportion [range], 31.2% [2.0%-33.7%] drug use vs 6.8% [0%-11.5%] drug use; *P* < .001) and had significantly higher pooled mean (range) triglyceride (233 [167-268] vs 171 [148-220] mg/dL; *P* = .01) and lower pooled mean (range) high-density lipoprotein cholesterol (40 [26-43] vs 46 [29-46] mg/dL; *P* = .03) levels. (To convert triglycerides and cholesterol to millimoles per liter, multiply by 0.0113 and 0.0259, respectively.) There were similar proportions of patients with diabetes, hypertension, and a family history of coronary artery disease in the 2 groups (Table 2; eTable 2 in Supplement 1).

**Table 2.** Patient Demographics With Breakdown of Total Number of Studies and Patients

Variable, %	Patients living with HIV			Patients without HIV			*P* value
	Studies, No. (N = 15)	Patients, No. (n = 9499)	Pooled mean (range)	Studies, No. (N = 15)	Patients, No. (n = 1 531 117)	Pooled mean (range)	
Follow up, mean, mo	14	9431	16.2 (3.0-60.8)	14	1 474 238	11.9 (3.0-60.8)	NA
Age, mean or median, y^a^	14	9020	56.2 (47.0-63.0)	14	1 529 553	67.7 (42.0-69.4)	<.001
Sex							
Male	14	9020	76.4 (64.3-100)	14	1 529 553	61.7 (59.7-100)	.94
Female	14	9020	23.6 (0-35.7)	14	1 529 553	38.3 (0-40.3)	.94
Race and ethnicity							
Black	5	7518	10.1 (7.0-62.5)	5	1 262 910	3.3 (2.5-21.4)	.60
Hispanic	3	6860	8.1 (0.4-54.6)	3	1 205 987	3.6 (0.7-36.3)	.73
White	4	7406	13.1 (7.2-64.0)	4	1 262 798	21.1 (14.3-68.0)	.94
Diabetes	14	9020	42.7 (8.7-50.7)	14	1 529 553	43.2 (10.7-47.8)	.98
Hypertension	14	9020	76.4 (17.4-87.3)	14	1 529 553	82.3 (22.1-89.3)	.79
Hyperlipidemia	10	1704	55.8 (25.0-84.2)	10	59 915	86.7 (29.0-88.6)	.07
Current smoker	11	7903	59.1 (24.0-75.0)	11	1 126 946	42.8 (26.0-64.1)	<.001
Illicit drug use	6	7472	31.2 (2.0-33.7)	6	1 176 953	6.8 (0-11.5)	<.001
CKD	8	8480	31.1 (2.1-35.6)	7	1 436 380	23.1 (1.8-25.6)	.67
Family history of CAD	8	1040	21.4 (13.5-56.3)	8	63 746	17.9 (15.9-59.4)	.77
BMI, mean	6	948	26.5 (22.0-29.7)	6	63 630	29.8 (26.0-30.4)	.27
Cholesterol, mean, mg/dL							
Total	7	549	193 (173-209)	7	5952	207 (177-210)	.09
HDL	7	549	40 (26-43)	7	5952	46 (29-46)	.03
LDL	6	416	111 (96- 133)	6	624	122 (107-136)	.21
Triglycerides, mean, mg/dL	6	416	233 (167-268)	6	624	171 (148-220)	.01
Diagnosis							
ACS	13	8474	99.0 (42.5-100)	12	1 472 694	100 (50.0-100)	.58
STEMI	13	8675	21.5 (9.2-100)	12	1 270 030	14.7 (6.3-100)	.63
NSTEMI	10	7933	49.2 (5.0-52.1)	9	1 267 550	50.8 (10.0-52.7)	.93
UA	8	7342	33.6 (17.4-45.5)	8	1 205 523	33.6 (12.5-50)	.99
Underwent PCI	12	8795	48.0 (35.3-100)	11	1 179 945	40.4 (30.9-100)	.83
Received stent	7	8017	32.3 (19.5-100)	7	1 177 361	24.4 (16.2-100)	.89
Received CABG	5	1292	0.8 (0-12.5)	4	59 363	0.1 (0-8.7)	.76
Discharged with medication							
Statin	7	7532	53.3 (45.8-96.1)	6	1 182 184	59.9 (58.4-99.0)	.79
β-blocker	5	7375	54.0 (51.3-90.0)	5	1 176 856	60.6 (59.6-93.6)	.74
Antiplatelet^b^	5	6962	39.1 (36.3-100)	5	1 125 373	43.2 (42.9-100)	.84
LVEF after event	8	1028	49.4 (44.0-55.4)	7	58 599	50.9 (48.0-54.8)	.20
HIV duration, mean, y	5	360	11.2 (8.5-12.0)	NA	NA	NA	NA
Current CD4 count, mean, cells/mm^3^	8	1025	377 (318-462)	NA	NA	NA	NA
Viral load <200 copies/mL	5	382	77.8 (63.3-94.6)	NA	NA	NA	NA
Prescribed ART	9	1246	75.2 (50.0-94.1)	NA	NA	NA	NA
Protease inhibitor							
Prescribed	8	1095	47.6 (25.0-85.6)	NA	NA	NA	NA
Duration, mean, mo	3	268	68.7 (36.0-81.0)	NA	NA	NA	NA

Abbreviations: ACS, acute coronary syndrome; ART, antiretroviral therapy; BMI, body mass index (calculated as weight in kilograms divided by height in meters squared); CABG, coronary artery bypass graft; CAD, coronary artery disease; CKD, chronic kidney disease; HDL, high-density lipoprotein; LDL, low-density lipoprotein; LVEF, left ventricular ejection fraction; NA; not applicable; NSTEMI, non–ST-segment elevation myocardial infarction; PCI, percutaneous coronary intervention; STEMI, ST-segment elevation myocardial infarction; UA, unstable angina.

SI conversion factors: To convert cholesterol to millimoles per liter, multiply by 0.0259; triglycerides to millimoles per liter, multiply by 0.0113.

^a^ Some studies provided median, which was taken as approximation of the central tendency, like the mean.

^b^ Parks et al^33^ defined antiplatelets as P2Y2 inhibitors, and the authors acknowledge that the study may have included a significant proportion of patients with type 2 myocardial infarction due to its retrospective, observational nature. When Parks et al^33^ is excluded for antiplatelet analysis, the aggregated percentage of patients discharged receiving antiplatelets was 92.8% (range, 86.7%-100%) for patients living with HIV and 97.0% (range, 81.5%-100%) for patients in control groups.

Patients with HIV had been diagnosed with HIV for a pooled mean (range) of 11.2 (8.5-12.0) years. From 9 studies^11,16,18,26,28,29,31,34,35^ that provided these data, a pooled proportion (range) of 75.2% (50.0%-94.1%) of patients living with HIV were receiving ART and 47.6% (25.0%-85.6%) had previously received protease inhibitor therapy. The pooled mean (range) CD4 count was 377 (318-462) cells/mm^3^ among patients living with HIV, and most of these patients (pooled proportion [range], 77.8% [63.3%-94.6%]) had a viral load less of than 200 copies per mL (Table 2).

Among 13 studies^11,15,16,18,25,26,27,28,29,31,32,33,34^ that reported data on ACS, patients living with HIV and those in control groups presented similarly with ST-segment elevation myocardial infarction, non–ST-segment elevation myocardial infarction, and unstable angina. Additionally, the groups received PCIs or coronary artery bypass graft surgery at similar proportions. After revascularization, pooled mean (range) left ventricular ejection fraction values were similar between groups (49.4% [44.0%-55.4%] vs 50.9% [48.0%-54.8%]). On postdischarge follow up, patients living with HIV had a lower proportion (range) of statin (53.3% [45.8%-96.1%] vs 59.9% [58.4%-99.0%]) and β-blocker (54.0% [51.3%-90.0%] vs 60.6% [59.6%-93.6%]) prescription compared with patients in control groups, but these differences were not statistically significant (Table 2; eTable 2 in Supplement 1).

**Figure 1.**
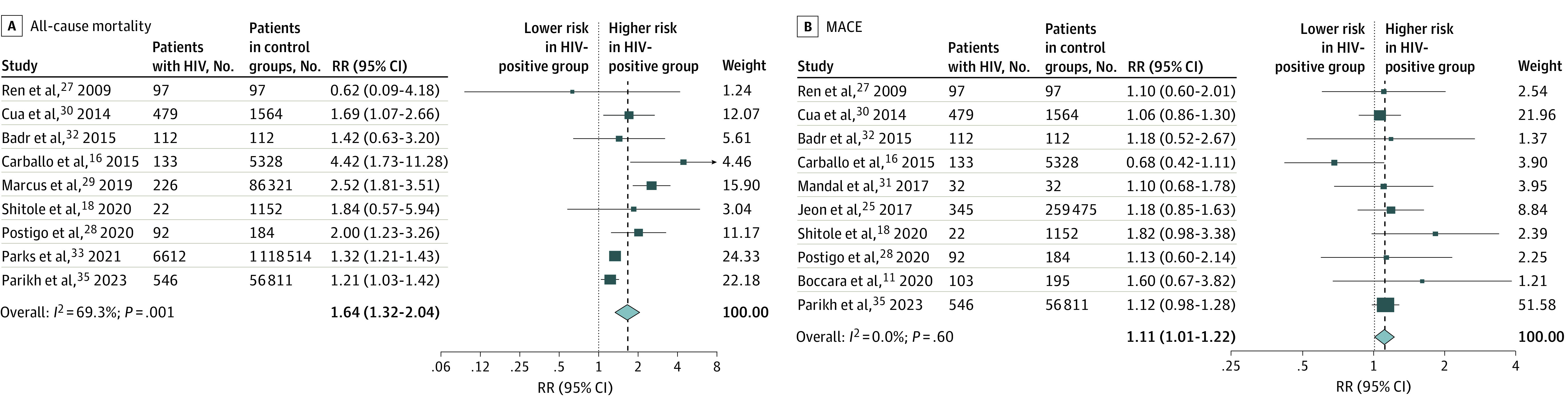
Pooled Relative Risks (RRs) for All-Cause Death and Major Adverse Cardiovascular Events (MACE) Dark blue boxes indicate RRs, and horizontal bars indicate 95% CIs. Sizes of dark blue boxes are proportional to the inverse variance. The light blue diamond indicates the pooled RR estimate and 95% CI in the random-effects model meta-analysis. RRs are maximally adjusted estimates as reported by studies (see eTable 1 in Supplement 1 for adjustment variables). Badr et al^32^ for RR of all-cause death and Postigo et al^28^ for RR of MACE were crude estimates calculated by this study’s authors based on number of participants and number of events reported for patients living with HIV and control groups. The definition of MACE for Shitole et al^18^ and Postigo et al^28^ was death or cardiovascular admissions.

**Figure 2.**
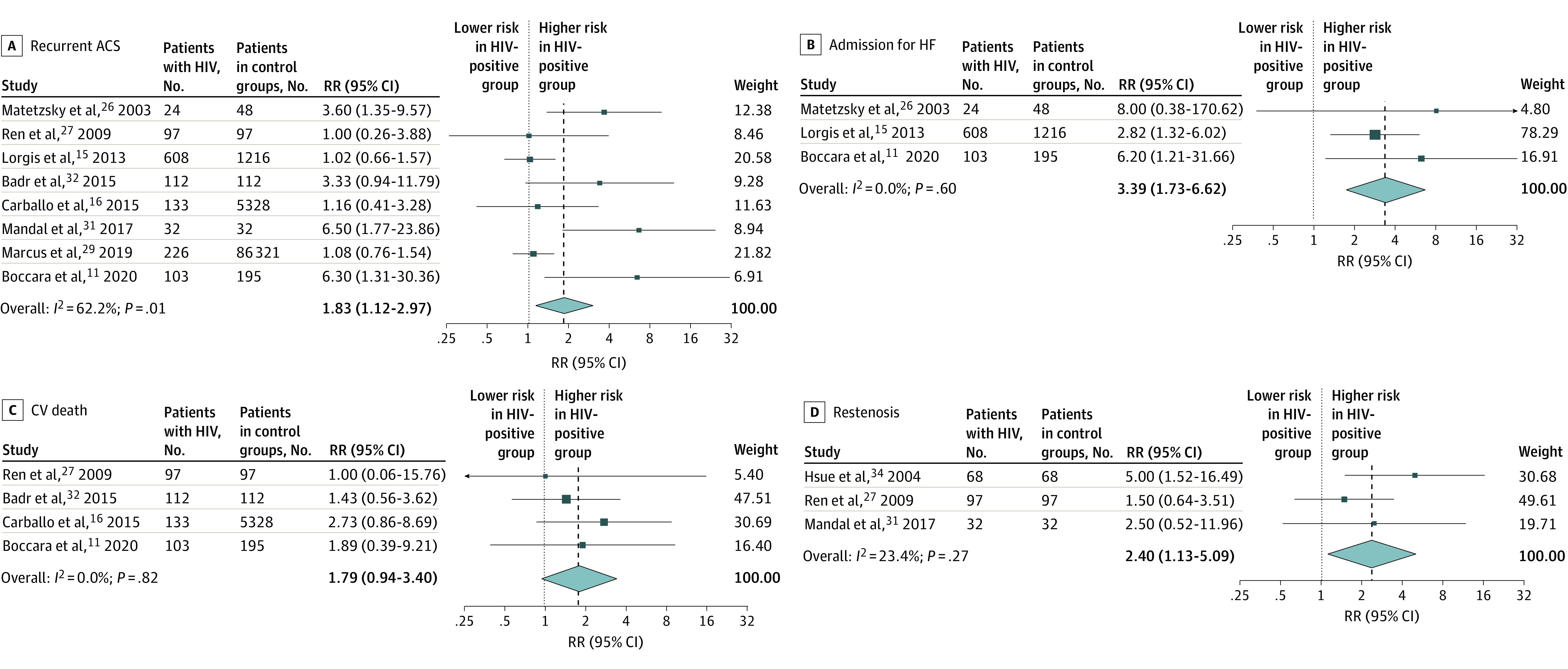
Pooled Relative Risks (RRs) for Other Outcomes RRs are shown for recurrent acute coronary syndrome (ACS) (A), heart failure (HF) admission (B), cardiovascular (CV) death (C), and restenosis (D). Dark blue boxes indicate RRs, and horizontal bars indicate 95% CIs. Sizes of dark blue boxes are proportional to the inverse variance. The light blue diamond indicates the pooled RR estimate and 95% CI in the random-effects model meta-analysis.

Over a pooled mean (range) follow-up of a mean of 16.2 (3.0-60.8) months after ACS or revascularization, patients living with HIV had a significantly higher adjusted risk of all-cause mortality (pooled adjusted RR, 1.64; 95% CI, 1.32-2.04), MACE (RR, 1.11; 95% CI, 1.01-1.22), recurrent ACS (RR, 1.83; 95% CI, 1.12-2.97), and heart failure readmission (RR, 3.39; 95% CI, 1.73-6.62) (Figure 1), as well as restenosis (RR, 2.40; 95% CI, 1.13-5.09) (Figure 2) compared with patients in HIV-negative control groups (pooled mean [range] follow-up, 11.9 [3.0-60.8] months). For CV death, total vessel revascularization, and total lesion revascularization, pooled HRs showed no significantly higher risk among patients living with HIV compared with patients in control groups (eFigure 2 in Supplement 1). RRs of clinical outcomes and adjustment variables included in multivariate models that were reported by each study are presented in eTable 3 in Supplement 1. Sensitivity analyses specifying an alternative method for the random-effects model yielded comparable results (eTable 4 in Supplement 1). In a separate subsidiary analysis, there was no association between HIV status and risk of post–ACS or PCI mortality, recurrent ACS, or MACE outcomes in the unadjusted (minimally adjusted in some studies) model (eFigure 3 in Supplement 1).

There was generally low heterogeneity across studies for most outcomes (Figure 1 and Figure 2). Visual inspection of the funnel plot for publication bias assessment and Egger tests did not suggest the presence of significant publication bias (eFigure 4 in Supplement 1). For the all-cause mortality outcome, the Egger test for bias was borderline, and so we performed trim and fill analysis; this yielded similar results (RR, 1.61; 95% CI, 1.30-2.00). Included studies were of moderate to high quality based on the Newcastle-Ottawa Scale, indicating a low to moderate risk of bias (eTable 5 in Supplement 1).

## Discussion

We performed a literature-based systematic review and meta-analysis of 15 studies of longitudinal clinical outcomes after ACS or revascularization from 2003 to 2023, comprising a total of 9499 patients living with HIV and 1 531 117 patients without HIV in control groups. We found that patients living with HIV were younger and had a higher risk of all-cause mortality, MACE, recurrent ACS, and heart failure after the index event. We also noted lower rates of statin and β-blocker prescription after discharge among patients living with HIV. Overall, these findings highlight the need to develop and implement strategies for secondary prevention of CVD among patients living with HIV.

The increased mortality, recurrence of ACS, and heart failure admissions among patients living with HIV may be attributed to increased traditional CVD risk factors, psychosocial factors, HIV-related chronic inflammation, and long-term effects of ART.^11,16^ These factors are equally difficult to control after an initial coronary event.^19,35,36^ The study by Boccara et al^11^ from 2020 compared its findings with those of their first, 2011 study^37^ and noted an increased rate of recurrence of ACS in patients living with HIV; the authors also noted persistent smoking and chronic inflammation as factors associated with some of the greatest increases in risk for recurrent disease. This further reinforces the need for a multifaceted approach to secondary prevention.

Of note, our study found suboptimal statin prescription in patients living with HIV after ACS or revascularization, which is consistent with results of other retrospective studies.^11,18,19,26,28,38,39,40,41,42^ These findings and those of the Evaluating the Use of Pitavastatin to Reduce the Risk of Cardiovascular Disease in HIV-Infected Adults (REPRIEVE) trial,^14^ which demonstrated the benefits of pitavastatin for primary prevention of atherosclerotic cardiovascular disease among patients living with HIV, highlight the need for a concerted effort to improve guideline-directed statin prescription and adherence among these patients.^43^ Additionally, the higher prevalence of smoking and higher triglyceride levels we found among patients living with HIV highlight areas for optimization, with the goal of improving secondary prevention of atherosclerotic cardiovascular disease. Differences in statin and β-blocker prescriptions on follow-up were not statistically significant, although patients living with HIV had numerically lower percentages for both outcomes.

Our pooled estimates for postdischarge antiplatelet therapy are influenced by the study from Parks et al,^33^ which defined antiplatelet use as a filled prescription for clopidogrel, ticagrelor, prasugrel, or ticlopidine and as a retrospective observational study, could not reliably exclude patients with type 2 myocardial infarctions who would not typically qualify for these therapies. In that study’s sensitivity analyses of patients who received coronary angiography, percentages of patients with postdischarge antiplatelet therapies were significantly higher. We performed an analysis of aggregate postdischarge antiplatelet therapy rates excluding data from Parks et al,^33^ and aggregate data for postdischarge antiplatelet therapy was much higher.

Few studies reported race or ethnicity of participants, leading to overall low aggregate percentages of White and Black patients living with HIV in our analysis, which is not representative of the global population of these patients. Race and ethnicity in most studies were obtained from review of electronic health records, except in the study by Shitole et al,^18^ in which race and ethnicity were self-reported. The analysis of race and ethnicity was skewed by 2 studies; in 1 study,^44^ most of the population’s race and ethnicity was unknown, and in the other study,^19^ the population was mainly Hispanic. Likewise, the percentage of patients who underwent PCIs was lower than expected for a typical population presenting with ACS. This was also contributed by the Parks et al study,^33^ which included patients with type 2 myocardial infarctions, who were not candidates for PCIs in their analysis.

Most studies in our analysis included patients receiving ART with low viral loads and CD4 counts greater than 200 cells/mm^3^, indicating patients with good control of their HIV disease, who are representative of people living with HIV in the current era.^1,4,7,45^ We found 8 studies^11,16,26,27,28,31,34,35^ that reported use of protease inhibitors among approximately 50% of patients living with HIV (47.6%). Protease inhibitors are known to have metabolic effects associated with CVD, presenting a plausible explanation for the difference in hypertriglyceridemia between patients living with HIV and patients without HIV in our study.^46^ Modern ART regimens have transitioned away from the use of protease inhibitors and now include integrase inhibitors.^7^ Conflicting data have emerged around the possible association of integrase inhibitors with increased incidence of CVD.^47,48^ Therefore, further research on long-term outcomes associated with ART will be essential to primary and secondary prevention of CVD among patients living with HIV.

The period after ACS or PCI provides additional opportunity to introduce aggressive interventions to improve CVD risk factors in patients living with HIV, and these interventions may involve multidisciplinary teams. Ensuring access to and engagement of cardiologists for patients living with HIV will be important to improve outcomes, especially among underrepresented racial and ethnic minorities.^49^ Input from pharmacists can also help with optimal selection of statin types, other lipid-lowering agents, and dosages to avoid drug interactions and drug-related adverse effects and maximize adherence to these therapies. Additionally, input from addiction medicine specialists and psychologists can help address underlying mental health disorders (eg, depression and anxiety) and behavioral risk factors (eg, smoking, alcohol use, and cocaine use). In our study, patients living with HIV were more likely to be smokers and engage in illicit drug use, similar to contemporary studies that also show that these behaviors are associated with an overall increased mortality in patients living with HIV despite adequate control of their underlying infection.^50^ Likewise, assistance from social workers can help to mitigate social determinants associated with diet and the ability to afford crucial medications.^36,51,52,53^ Addressing this latter aspect is critically important to improve secondary outcomes of CVD in patients living with HIV because despite increased prescription rates for cardioprotective medications, patients living with HIV have been found to be less likely to fill these medications.^38,42,52^ A multifaceted or multidisciplinary intervention to address psychosocial barriers to cardiovascular care may have the potential to limit mortality and morbidity after ACS or PCI for patients living with HIV.

### Limitations

The findings of this meta-analysis should be considered in context of several limitations. First, given that this was a literature based meta-analysis of aggregate published data, we were unable to compare the association between HIV status and CVD outcomes by clinically important subgroup, such as age, race and ethnicity, or sex. Second, the degree of adjustment for confounders in RR estimates is limited to what is reported in individual studies, is not consistent across studies, and may be inadequate overall. For instance, very few studies accounted for HIV-specific characteristics. However, the goal of the meta-analysis was to understand the difference in secondary CVD outcomes stratified by HIV status regardless of factors that may be contributing to them. We also performed a comparison between maximally adjusted and unadjusted or minimally adjusted RRs to provide further insight into the association. Our analysis showed that there was no association between HIV status and post-ACS or -PCI mortality, recurrent ACS, or MACE outcomes in the unadjusted model. This is likely due to the reverse confounding effect of age given that patients living with HIV were significantly younger than patients in control groups, with a difference of 11 years in pooled mean age across studies. Third, most studies included in this review evaluated patients living with HIV who lived in high-income countries, which may limit generalizability to the global population of patients living with HIV. Fourth, we were not able to perform subgroup analyses of patients who had ACS and were treated medically vs PCI, as well as those who received PCI for stable coronary disease, because these data were not reported separately. Future assessment of outcomes within these subgroups would be important for preventative efforts. Fifth, we were unable to identify timelines for prescription of or adherence to ART or cardioprotective medications based on these aggregate data. Understanding these trends will also be an important focus for secondary prevention in future studies.

## Conclusions

In this literature based systematic review and meta-analysis of longitudinal studies from 2000 to 2023, we found that patients living with HIV were significantly younger than patients in control groups. Patients living with HIV had a significantly higher risk of all-cause mortality, MACE, recurrent ACS, and admission for heart failure after the index event compared with patients in control groups.

Patients living with HIV were also significantly more likely to be current smokers and engage in illicit drug use and had higher triglyceride levels at baseline. As more data emerge for primary prevention, this analysis highlights the need for optimization of secondary prevention strategies to address poor outcomes of CVD among patients living with HIV. Future studies can focus on assessing the role of aggressive interventions, including use of multidisciplinary teams to target important risk factors and improve prescription of and adherence to cardioprotective medications among patients living with HIV after ACS or PCI.

## Notes

Supplement 1. **eTable 1.** Additional Patient Characteristics by Study for Patients Living With HIV and Patients in Control Groups **eTable 2.** Comparison of Patient Characteristics Between Patients Living With HIV and Patients in Control Groups **eTable 3.** Clinical Outcomes, Relative Risks, and Adjustment Variables by Study **eTable 4.** Sensitivity Analysis of Pooled Relative Risks Calculated Using Knapp-Hartung Method for Random-Effects Model Meta-Analysis **eTable 5.** Quality Assessment of Included Studies With Newcastle-Ottawa Scale **eFigure 1.** Study Flow Sheet **eFigure 2.** Pooled Relative Risks for Patients Living With HIV vs Patients in Control Groups for TLR and TVR **eFigure 3.** Pooled Unadjusted Relative Risks for Patients Living With HIV vs Patients in Control Groups for All-Cause Mortality, MACE, and Recurrent ACS **eFigure 4.** Funnel Plot of Relative Risks for All-Cause Mortality and MACE **eMethods.** Detailed Description of Statistical Analysis

Supplement 2.
Data Sharing Statement
