# Supplementary material for: Clinical Outcomes After Acute Coronary Syndromes or Revascularization Among People Living With HIV: A Systematic Review and Meta-Analysis
Source: JAMA Netw Open. 2024 May 14;7(5):e2411159. doi: 10.1001/jamanetworkopen.2024.11159 (PMC11094563; doi:10.1001/jamanetworkopen.2024.11159)
Supplement: Supplement 1. — eTable 1. Additional Patient Characteristics by Study for Patients Living With HIV and Patients in Control Groups eTable 2. Comparison of Patient Characteristics Between Patients Living With HIV and Patients in Control Groups eTable 3. Clinical Outcomes, Relative Risks, and Adjustment Variables by Study eTable 4. Sensitivity Analysis of Pooled Relative Risks Calculated Using Knapp-Hartung Method for Random-Effects Model Meta-Analysis eTable 5. Quality Assessment of Included Studies With Newcastle-Ottawa Scale eFigure 1. Study Flow Sheet eFigure 2. Pooled Relative Risks for Patients Living With HIV vs Patients in Control Groups for TLR and TVR eFigure 3. Pooled Unadjusted Relative Risks for Patients Living With HIV vs Patients in Control Groups for All-Cause Mortality, MACE, and Recurrent ACS eFigure 4. Funnel Plot of Relative Risks for All-Cause Mortality and MACE eMethods. Detailed Description of Statistical Analysis [file jamanetwopen-e2411159-s001.pdf]

Supplemental Online Content

Haji M, Capilupi M, Kwok M, et al. Clinical outcomes after acute coronary syndromes or revascularization among people LIving with HIV: a systematic review and meta-analysis. *JAMA Netw Open*. 2024;7(5):e2411159. doi:10.1001/jamanetworkopen.2024.11159

eTable 1. Additional Patient Characteristics by Study for Patients Living With HIV and Patients in Control Groups
eTable 2. Comparison of Patient Characteristics Between Patients Living With HIV and Patients in Control Groups
eTable 3. Clinical Outcomes, Relative Risks, and Adjustment Variables by Study
eTable 4. Sensitivity Analysis of Pooled Relative Risks Calculated Using Knapp-Hartung Method for Random-Effects Model Meta-Analysis
eTable 5. Quality Assessment of Included Studies With Newcastle-Ottawa Scale
eFigure 1. Study Flow Sheet
eFigure 2. Pooled Relative Risks for Patients Living With HIV vs Patients in Control Groups for TLR and TVR
eFigure 3. Pooled Unadjusted Relative Risks for Patients Living With HIV vs Patients in Control Groups for All-Cause Mortality, MACE, and Recurrent ACS
eFigure 4. Funnel Plot of Relative Risks for All-Cause Mortality and MACE
eMethods. Detailed Description of Statistical Analysis

This supplemental material has been provided by the authors to give readers additional information about their work.

© 2024 Haji M et al. *JAMA Network Open*.

eTable 1A: Additional Patient Characteristics by Study for Patients Living with HIV.

Author	Publication year	n	Male (%)	White (%)	Black (%)	Hispanic (%)	Av. Age (yrs) *	DM (%)	HTN (%)	HLD (%)	Cur. Smoker (%)	CKD (%)	FHx CAD (%)	Av. BMI (kg/m2)	ACS (%)	STE MI (%)	NSTE MI (%)	UA (%)	PCI (%)	Statin on DC (%)	BB on DC (%)	Av. HIV Dur. (yrs) *	ART (%)	Cur. CD4 count*	VL < 200 copies (%)	PI (%)
Matetzsky	2003	24	87.5	-	-		47 (9)	12.5	29.1	58.3	58.3	-	50.0	-	100	58.3	41.7	-	70.8	45.8	-	10 (4)	91.7	318 (210)	-	70.8
Hsue	2004	68	89.7	-	-		50	13.2	52.9	25.0	67.6	14.7	35.2	-	99.9	29.4	25	45.5	43	-	-	8.5	52.9	341	-	48.5
Ren	2009	97	100	-	-		53 (9)	10	46	60	24	10	-	-	80	21	26	33	100	-	-	-	-	406	-	81
Lorgis	2013	608	88.6	-	-		50 (10)	9.1	17.4	31.1	29.6	2.1	-	-	100	90.5	-	-	66.4	-	-	-	-	-	-	-
Carballo	2015	133	85.0	-	-		51 (46-67)	13.5	24.1	-	58.3	-	13.5	23.0	100	48.2	51.8	-		86.5	-	12 (8-18)	90.2	462 (347-401)	77.2	85.6
Badr	2015	112	64.3	-	62.5		58.0 (11.4)	24.5	84.8	78.6	30.4	27.7	42.9	29.7	59.4	18.9	-	40.5	100	-	-	-	-	-	-	-
Jeon	2017	345	87.0	-	-		54.4 (10.5)	18.0	37.4	-	-	11.9	-	-	100	-	-	-		-	-	-	-	-	-	-
Mandal	2017	32	93.8	-	-		49 (7.1)	21.9	28.1	46.9	75.0	-	56.3	22	42.5	12.5	5.0	25.0	100	-	-	12 (7.2)	50.0	412 (260-620)	71.9	25.0
Cua	2018	479		-	-		-	-	-	-	-	-	-	-	-	-	-	-	100	-	-	-			-	-
Marcus	2019	226	94	64	15	0.4	54 (9.3)	21	53	-	-	-	-	-	100	34	36	30		-	64	-	84		-	-
Boccara	2020	103	93.2	-	-		48 (9.1)	8.7	18.4	39.8	59.2	-	20.4	22.0	100	48.5	20.4	31.1	76.2	96.1	56.3	12 (7.9)	94.1	462 (347-401)	66.0	82.5
Shitole	2020	22	72.7	9.1	22.7	54.6	50 (43-57)	22.7	59.1	50.0	68.2	-	27.3	25.7	100	100	-	-	90.9	90.0	90.0	-	70.0	372 (135-570)	63.3	-
Postigo	2020	92	92.4	-	-		51.3 (9)	14.1	41.3	57.6	71.7	12	-	-	100	46.7	35.9	17.4	84.1	86.7	75.6	-	92.4		94.6	41.2
Parks	2021	6612	71.2	7.2	7	8.2	57.4 (10.5)	50.7	87.3	-	62.5	35.6	-	-	100	14.1	52.1	33.8	35.3	48.5	51.3	-	-	-	-	-
Parikh	2023	546	98.9	63.4	34.8		63 (9.4)	42.3	86.8	84.2	-	30.4	14.3	27.7		9.2	37.2	-	100	88.1	81.0	-	64.4	341	-	26.6

Av. – average, BB – beta-blocker, BMI – body mass index, CKD – chronic kidney disease, Cur – current, DC – discharged, DM – diabetes mellitus, Dur – duration, FHx – family history, HLD – hyperlipidemia, HTN – hypertension, MI – myocardial infarction, NSTE – non-ST elevation, PCI – percutaneous coronary intervention, PI – protease inhibitor, STEMI – T-elevation MI, VL – viral load.

*Values in parentheses represents standard deviation for studies that provided averages or interquartile range for the studies that provided medians

© 2024 Haji M et al. *JAMA Network Open*.

eTable 1B: Additional Patient Characteristics by Study for Patients in Control Groups.

Author	Publication year	n	Male (%)	White (%)	Black (%)	Hispanic (%)	Av. Age (years) *	DM (%)	Hypertension (%)	HLD (%)	Current Smoker (%)	CKD (%)	Fam History CAD (%)	Av. BMI (kg/m2)	ACS (%)	STEMI (%)	NSTEMI (%)	Unstable Angina (%)	PCI (%)	Statin on discharge (%)	BB on Discharge (%)
Matetzsky	2003	48	87.5	-	-		48 (7)	18.8	43.7	56.3	47.9	-	43.8	-	-	-	-	-	-	-	-
Hsue	2004	68	61.7	-	-		61	41.1	60.3	41.2	41.2	13.2	23.5	-	100	34.3	36.8	27.9	30.9	-	-
Ren	2009	97	100	-	-		54 (9)	26	67	65	26	-	-	-	79	19	10.0	50	100	-	-
Lorgis	2013	1216	88	-	-		50 (9.5)	10.7	22.1	29.0	30.2	1.8	-	-	100	91.0	-	-	61.7	-	-
Carballo	2015	5328	72.2	-	-		64 (55-74)	15.6	60.7	-	39.4		33.3	26.5	100	58.8	41.2	-		94.4	-
Badr	2015	112	64.3	-	21.4		58.0 (11.4)	24.5	83.0	80.9	26.8	9.9	54.3	30.4	64.8	24.3	-	40.5	100	-	-
Jeon	2017	259475	61.7	-	-		69.4 (14.3)	31.8	66.0	-	-	12.5	-	-	100	-	-	-		-	-
Mandal	2017	32	87.5	-	-		42 (7.4)	31.3	34.8	53.1	62.5	-	59.4	26.0	50.0	6.3	31.3	12.5	100	-	-
Cua	2018	1564		-	-		-	-	-	-	-	-	-	-	-	-	-	-	100	-	-
Marcus	2019	86321	63	68	6.5	0.7	67 (13)	29	72	-	-	-	-	-	100	21	38	42		-	-
Boccara	2020	195	94.3	-	-		50 (9.5)	11.8	24.1	43.1	64.1	-	27	27	100	55,9	21.0	23.1	86.2	99.0	61.0
Shitole	2020	1152	66.7	23.1	20.3	36.3	60 (51-70)	33.4	67.1	54.1	36.8	-	30.6	28.2	100	100	-	-	95.2	96.7	93.6
Postigo	2020	184	92.4				51.3 (9)	20.7	38.6	42.9	64.1	3.3	-	-	100	44.6	42.9	12.5	82.1	96.7	87.4
Parks	2021	1118514	59.7	14.3	2.5	3.8	67.4 (12.9)	46.9	86.8	-	42.8	25.6	-	-	100	14.3	52.7	33	37.2	58.4	59.6
Parikh	2023	56811	98.1	83.7	14.0		67.1 (9.4)	47.8	89.3	88.6	-	21.5	15.9	30.2		5.9	33.3	-	100	84.9	78.7

Av. – average, BB – beta-blocker, BMI – body mass index, CKD – chronic kidney disease, Cur – current, DC – discharged, DM – diabetes mellitus, Dur – duration, FHx – family history, HLD – hyperlipidemia, HTN – hypertension, MI – myocardial infarction, NSTE – non-ST elevation, PCI – percutaneous coronary intervention, PI – protease inhibitor, STEMI – T-elevation MI, VL – viral load.

*Values in parentheses represents standard deviation for studies that provided averages or interquartile range for the studies that provided medians

© 2024 Haji M et al. *JAMA Network Open*.

eTable 2: Comparison of Patient Characteristics Between Patients Living with HIV and Patients in Control Groups.

Variable	N	Delta*	95% LCL	95% UCL	SE	P-Value
Age (y)	28	-11.08	-15.96	-6.20	2.37	p < 0.001
Male (%)	28	14.63	-23.97	53.24	18.78	0.94
White (%)	8	-8.16	-259.94	243.61	102.89	0.94
Black (%)	10	6.83	-21.75	35.40	12.39	0.60
Hispanic (%)	6	7.47	-49.15	64.08	20.39	0.73
Diabetes (%)	28	-0.51	-37.45	36.44	17.97	0.98
Hypertension (%)	28	-5.91	-51.76	39.94	22.31	0.79
Hyperlipidemia (%)	20	-30.91	-64.15	2.33	15.82	0.07
Current Smoker (%)	22	16.36	10.17	22.55	2.97	p < 0.001
Illicit Drug Use (%)	12	24.20	16.80	32.10	3.40	p < 0.001
CKD (%)	15	8.02	-32.00	48.04	18.53	0.67
Family History of CAD (%)	16	3.58	-22.21	29.37	12.02	0.77
Body mass index (kg/m^2)	12	-3.36	-9.79	3.08	2.89	0.27
Total cholesterol (mg/dL)	14	-13.76	-29.84	2.32	7.38	0.09
HDL-cholesterol (mg/dL)	14	-6.07	-11.45	-0.69	2.47	0.03
LDL-cholesterol (mg/dL)	12	-10.35	-27.61	6.90	7.74	0.21
Triglycerides (mg/dL)	12	61.47	17.28	105.65	19.83	0.01
Diagnosed with ACS (%)	25	-0.98	-4.57	2.61	1.73	0.58
Diagnosed with STEMI (%)	25	6.79	-21.88	35.45	13.86	0.63
Diagnosed with NSTEMI (%)	19	-1.51	-36.76	33.74	16.71	0.93
Diagnosed with UA (%)	16	-0.05	-17.40	17.29	8.09	0.99
Underwent PCI (%)	23	7.58	-65.95	81.11	35.36	0.83
Received Stent (%)	14	7.84	-110.67	126.35	54.39	0.89
Received CABG (%)	9	0.71	-4.47	5.89	2.19	0.76
Discharged with statin (%)	13	-6.65	-59.79	46.49	24.15	0.79
Discharged with beta blocker (%)	10	-6.60	-51.52	38.31	19.48	0.74
Discharged with antiplatelet (%)	10	-4.10	-49.07	40.88	19.50	0.84
Post-EF (%)	15	-1.47	-3.82	0.88	1.09	0.20

***Mean difference between PLWH vs. patients in control groups**

ACS – acute coronary syndrome, CABG -coronary artery bypass graft, CAD -coronary artery disease, CKD – chronic kidney disease, EF – ejection fraction, HDL – high density lipoprotein, LCL – lower confidence limit, LDL – low density lipoprotein, N – number of studies, NSTEM – non-ST elevation myocardial infarction, PCI -percutaneous coronary angiography, PLWH -patients living with HIV, SE -standard error, STEMI – ST elevation myocardial infarction, UA – unstable angina, UCL – upper confidence limit.

© 2024 Haji M et al. *JAMA Network Open*.

eTable 3: Clinical Outcomes, Relative Risks, and Adjustment Variables by Study.

Study	Events HIV	Events Control	n HIV	n Control	Relative Risks	Adjustment Variables
**CV Death**						
Boccara_2020	3	3	103	195	1.89 (0.39-9.21)	IDU, BMI (≥ versus <25 kg/m2), LDL (> versus ≤100 mg/dL), TG (> versus ≤150 mg/dL)
Ren_2009	1	1	97	97	1.00 (0.06-15.76)	Age, HIV characteristics (CD4 count, protease inhibitor treatment), CAD risk factors (HTN, dyslipidemia, DM, family hx, current tobacco use, cocaine or amphetamine use, CAD hx, CABG, CKD), medications before admission (Aspirin, beta blockers, ACEi, CCB, statin), indications for PCI (STEMI, NSTEMI, UA, stable angina), lesion characteristics (no. of narrowed coronary arteries, culprit lesion in LAD, bifurcation lesion), stent characteristics (no. of stents, stent diameter, total stent length), lipid panel (total cholesterol, TG, HDL, LDL), LVEF, SBP, DBP, HR, creatinine, periprocedural glycoprotein IIb/IIIa use
Carballo_2015	3	44	133	5328	2.73 (0.86-8.69)	Age, sex, calendar year of AMI, smoking status, hypertension and diabetes
Badr_2015	10	7	112	112	1.43 (0.56-3.62)	Black race, CKD, and acute MI as initial diagnosis
**All-Cause Mortality**						
Shitole_2020	3	185	22	1152	1.84 (0.57-5.94)	Age, sex and race-ethnicity, BMI, SES score, current smoking, heavy alcohol use and cocaine use, diabetes, hypertension, prior ASCVD, prior HF, initial creatinine, Killip class, LVEDP, LHC within 24 hours of presentation and CABG during the index hospitalization.
Ren_2009	3	2	97	97	0.62 (0.09-4.18)	Same as CV death adjustment
Postigo_2020	6	7	92	184	2.00 (1.23-3.26)	Age, previous CAD, creatinine, leukocyte count

© 2024 Haji M et al. *JAMA Network Open*.

Carballo_2015	5	135	133	5328	4.42 (1.73-11.28)	Age, sex, calendar year of AMI, smoking status
Marcus_2019	35	16401	226	86321	2.52 (1.81-3.51)	Age, sex, race/ethnicity (white, black, or other), ACS hospitalization prior year, ACS type (STEMI, NSTEMI or unstable angina), smoking (ever or never), LDL (≥ 160 or < 160 mg/dL), HDL (< 40 or ≥ 40 mg/dL), TG (< 150 or ≥ 150 mg/dL), statin prior year to ACS, HTN, DM prior year to ACS hospitalization.
Cua_2018	35	73	479	1564	1.69 (1.07-2.66)	Age, gender, race/ethnicity, hypertension, diabetes, dyslipidemia, smoking, hepatitis C, renal disease, anemia, and substance use.
Badr_2015	17	12	112	112	1.42 (0.63-3.20)	Same as CV death adjustment
Parks_2021 **	724	114933	6612	1.1e+06	1.32 (1.21-1.43)	Age, sex, medical comorbidities, and ACS sub-type
Parikh_2023	57	4373	546	56811	1.21 (1.03-1.42)	Age, Race, Prior PCI, Presentation, HIV Related Factors (viral load, ART use), Procedural Factors (extent of CAD, access site, hospital volume)
			**<u>TVR</u>**			
Boccara_2020	12	22	103	195	1.01 (0.47-2.19)	Same as CV death adjustment
Ren_2009	22	20	97	97	1.10 (0.52-2.31)	Same as CV death adjustment
Mandal_2017	2	1	32	32	1.60 (0.60-4.28)	-
Badr_2015	12	8	112	112	1.50 (0.64-3.53)	Black race, CKD, and acute MI as initial diagnosis
			**<u>TLR</u>**			
Boccara_2020	9	18	103	195	0.80 (0.33-1.93)	Same as CV death adjustment
Ren_2009	17	13	97	97	1.30 (0.58-2.91)	Same as CV death adjustment
Mandal_2017	2	1	32	32	1.40 (0.74-2.66)	-
Badr_2015	8	8	112	112	1.00 (0.39-2.57)	Same as CV death adjustment
			**<u>Restenosis</u>**			

© 2024 Haji M et al. *JAMA Network Open*.

Ren_2009	12	8	97	97	1.50 (0.64-3.51)	Same as CV death adjustment
Mandal_2017	5	2	32	32	2.50 (0.52-11.96)	-
Hsue_2004	15	3	68	68	5.00 (1.52-16.49)	***
**Recurrent Revascularization**						
Boccara_2020	15	27	103	195	1.06 (0.51-2.22)	Same as CV death adjustment
Lorgis_2013 **	14	18	608	1216	1.56 (0.78-3.11)	***
**Recurrent ACS**						
Boccara_2020	14	15	103	195	6.30 (1.31-30.36)	Same as CV death adjustment
Lorgis_2013 **	29	67	608	1216	1.02 (0.66-1.57)	History of ischemic cardiomyopathy
Matetzsky_2003	9	5	24	48	3.60 (1.35-9.57)	***
Ren_2009	4	4	97	97	1.00 (0.26-3.88)	Same as CV death adjustment
Carballo_2015	4	146	133	5328	1.16 (0.41-3.28)	Same as CV death adjustment
Marcus_2019	34	13799	226	86321	1.08 (0.76-1.54)	Same as All-cause mortality
Mandal_2017	5	2	32	32	6.50 (1.77-23.86)	-
Badr_2015	10	3	112	112	3.33 (0.94-11.79)	Same as CV death adjustment
**MACE**						
Boccara_2020	22	39	103	195	1.60 (0.67-3.82)	Same as CV death adjustment
Shitole_2020	11	425	22	1152	1.82 (0.98-3.38)	Same as All-cause mortality
Jeon_2017			345	259475	1.18 (0.85-1.63)	Age, sex, neighborhood income, urban vs. rural, comorbidity burden in past year, cardiology visit in past year, hx of DM, HTN, COPD, CHF at any time prior to presenting MI.
Ren_2009	32	29	97	97	1.10 (0.60-2.01)	Same as CV death adjustment
Postigo_2020	17	30	92	184	1.13 (.60-2.14)	Same as All-cause mortality
Carballo_2015	20	1078	133	5328	0.68 (0.42-1.11)	Same as CV death adjustment
Cua_2018	143	451	479	1564	1.06 (0.86-1.30)	Age, gender, race/ethnicity, hypertension, diabetes, dyslipidemia, smoking, hepatitis C,

© 2024 Haji M et al. *JAMA Network Open*.

						renal disease, anemia, and substance use.
Mandal_2017	3	1	32	32	1.10 (0.68-1.78)	-
Badr_2015	28	19	112	112	1.18 (0.52-2.67)	Same as CV death adjustment
Parikh_2023	121	9910	546	56811	1.12 (0.98-1.28)	Same as All-cause mortality
**HF Admission**						
Boccara_2020	12	3	103	195	6.20 (1.21-31.66)	Same as CV death adjustment
Lorgis_2013 **	15	14	608	1216	2.82 (1.32-6.02)	HIV infection, diabetes mellitus, history of ischemic CMP, and PCI
Matetzsky_2003	2	0	24	48	8.00 (0.38-170.62)	***

** Indicates studies that used Odds Ratios instead of Hazard Ratios: Lorgis_2013 and Park_2021
*** represents crude risk ratios calculated by us using numbers provided in the publication.

- Indicates studies that reported unadjusted risk ratios.

Abbreviations: ACEi – angiotensin converting enzyme inhibitors, AMI – acute myocardial infarction, ART – antiretroviral therapy, ASCVD – atherosclerotic cardiovascular disease, BMI – body mass index, CABG – coronary artery bypass graft, CAD – coronary artery disease, CCB – calcium channel blocker, CKD – chronic kidney disease, CMP -cardiomyopathy, COPD – chronic obstructive pulmonary disease, CV – cardiovascular, DBP -diastolic blood pressure, DM – diabetes mellitus, HDL – high-density lipoprotein, HF -heart failure, HR – heart rate, HTN – hypertension, IDU – illicit drug use, LAD – left anterior descending coronary artery, LHC – left heart catheterization, LDL – low-density lipoprotein, LVEDP – left ventricular end diastolic pressure, LVEF – left ventricular ejection fraction, PCI -percutaneous coronary angiography, MI – myocardial infarction, NSTEMI – non-ST elevation myocardial infarction, SBP – systolic blood pressure, SES – socioeconomic status, STEMI – ST elevation myocardial infarction, TG – triglycerides, TLR – target lesion revascularization, TVR – target vessel revascularization, UA – unstable angina

© 2024 Haji M et al. *JAMA Network Open*.

eTable 4: Sensitivity Analysis of Pooled Relative Risks Calculated using Knapp-Hartung Method for Random-Effects Model Meta-analysis.

	RR	SE	z-value	p-value
Death	1.64 (1.23 – 2.19)	0.20	4.00	0.002
MACE	1.11 (0.99 – 1.24)	0.05	2.09	0.067
Recurrent ACS	1.67 (0.95- 2.94)	0.41	2.08	0.071
Admission for Heart Failure	3.39 (0.78- 14.76)	1.16	3.57	0.07
CV Death	1.79 (0.63 – 5.07)	0.59	1.78	0.173
Re-stenosis	2.43 (0.42- 14.04)	0.99	2.18	0.161
TLR	1.16 (0.61-2.20)	0.23	0.73	0.519
TVR	1.23 (0.63 – 2.4)	0.26	0.99	0.394
Recurrent Revascularization	1.90 (0.06 -3.67)	0.53	2.3	0.055

RR – relative risk, SE-standard error

© 2024 Haji M et al. *JAMA Network Open*.

eTable 5: Quality Assessment of Included Studies with Newcastle-Ottawa Scale

	Selection				Comparability		Outcome			
Study	Representative of the exposed cohort	Selecti of the external control	Ascertainment of exposure	Outcome of interest not present at the start of the study	Main factor	Additional factor	Assessment of outcomes	Sufficient follow-up time	Adequacy of follow up	Total
Matetzsky 2003	*	*	*	*	*	*	*	*	*	9
Hsue 2004	*	*	*	*	*	*	*			7
Ren 2009	*	*	*	*	*	*	*	*	*	9
Boccara 2011	*	*	*	*	*	*	*	*	*	9
Llorgis 2013	*	*	*	*	*	*	*	*		8
Carballo 2015	*	*	*	*	*	*	*	*	*	9
Badr 2015	*	*	*	*	*	*	*	*		8
Mandal 2017	*	*	*	*	*	*	*	*	*	9
Cua 2018	*	*	*	*	*	*	*	*		8
Marcus 2019	*	*	*	*	*	*	*	*		8
Boccara 2020	*	*	*	*	*	*	*	*	*	9
Shitole 2020	*	*	*	*	*	*	*	*		8
Postigo 2020	*	*	*	*	*	*	*	*		8
Parks 2021	*	*	*	*	*	*	*	*		8
Parikh 2023	*	*	*	*	*	*	*	*		8

© 2024 Haji M et al. *JAMA Network Open*.

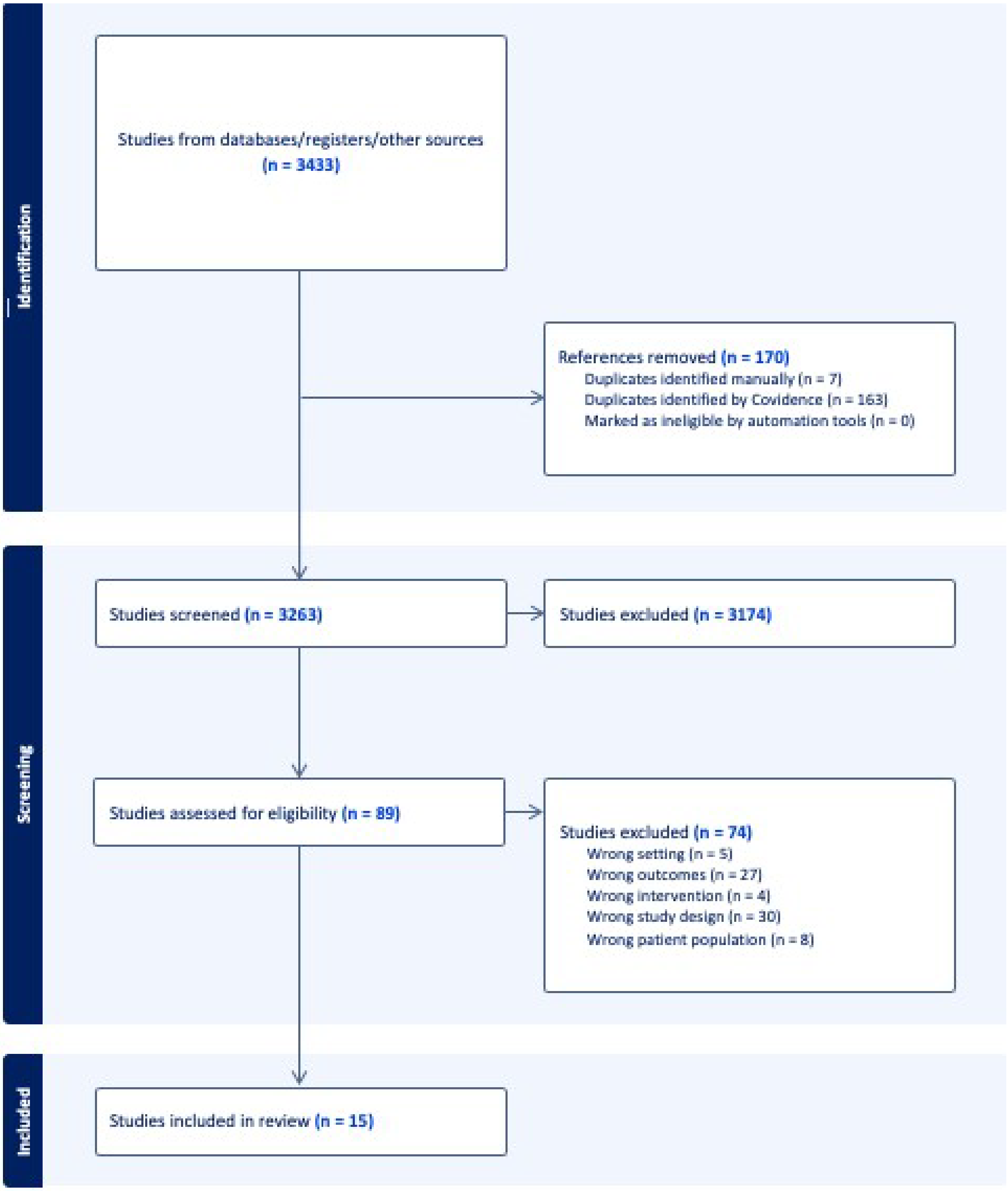

eFigure 1. Study Flowsheet.

© 2024 Haji M et al. *JAMA Network Open*.

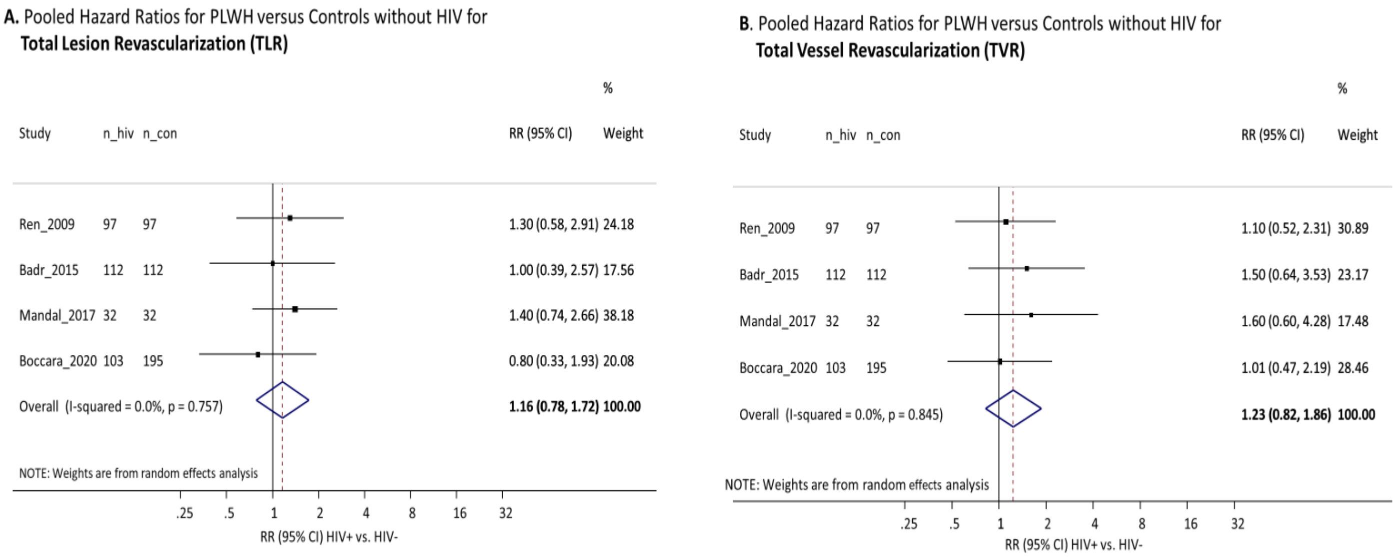

eFigure 2: Pooled Hazard Ratios for PLWH versus Controls without HIV for **TLR (A), TVR (B)** outcomes

© 2024 Haji M et al. *JAMA Network Open*.

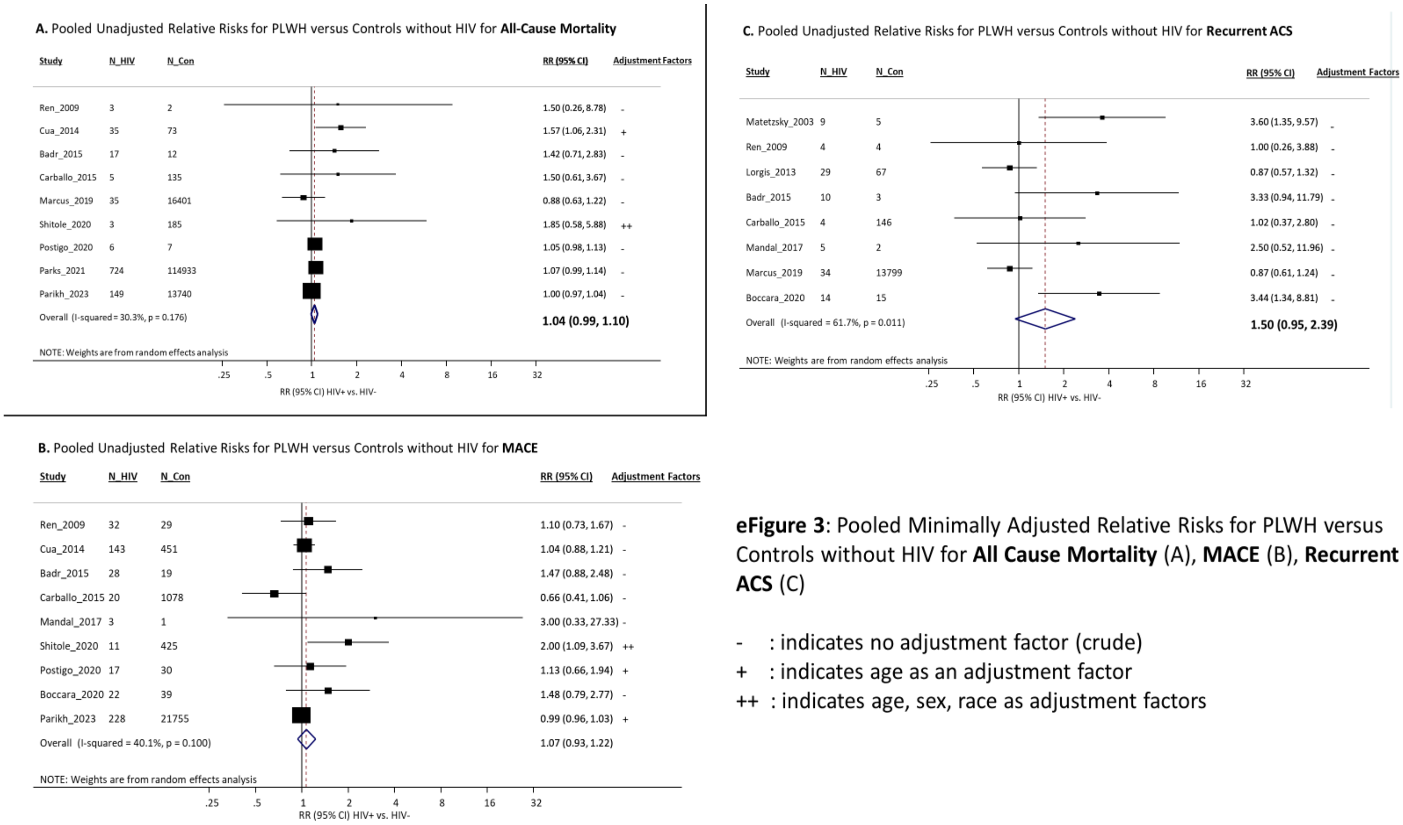

eFigure 3: Pooled Minimally Adjusted Relative Risks for PLWH versus Controls without HIV for **All Cause Mortality** (A), **MACE** (B), **Recurrent ACS** (C)

- : indicates no adjustment factor (crude)
+ : indicates age as an adjustment factor
++ : indicates age, sex, race as adjustment factors

© 2024 Haji M et al. *JAMA Network Open*.

(A) All-cause mortality (B) MACE

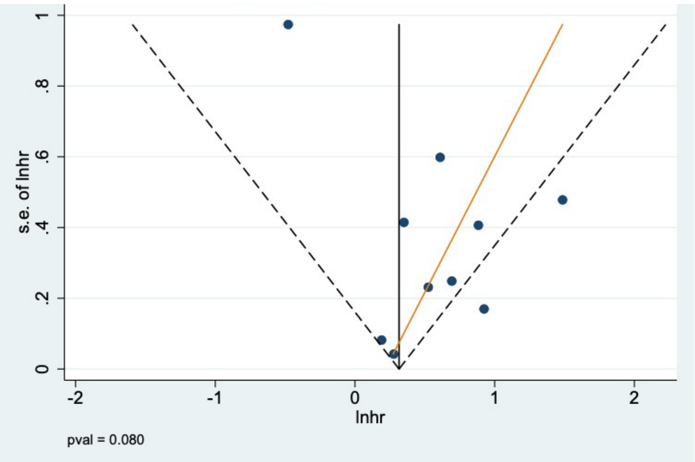

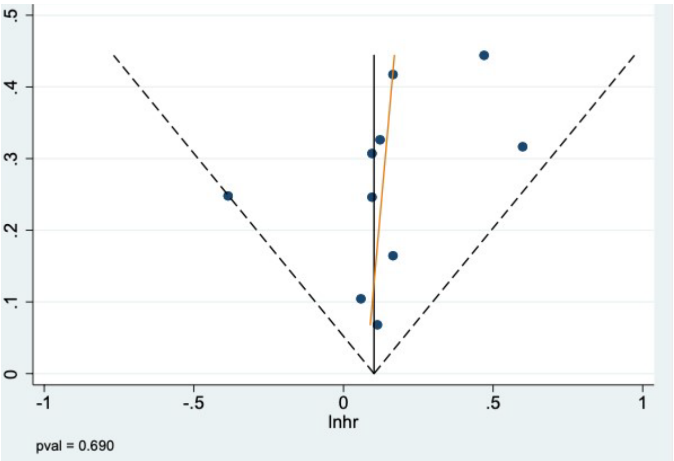

eFigure 4: Funnel Plot of Hazard Ratios for (A) All-Cause Mortality, and (B) MACE.

© 2024 Haji M et al. *JAMA Network Open*.

eMethods: Detailed Description of the Meta-analytic Methods

The flow sheet for search strategy is presented in **eFigure 1**. The data sought and extracted using a standardized form is presented in **Table 1** and **eTables 1** and **3**. In addition to study characteristics and baseline characteristics of participants, where available, we extracted relative risk (RR) estimates and number of events within each group for all-cause mortality, major adverse cardiovascular event (MACE), recurrent acute coronary syndrome (ACS), recurrent heart failure admission, cardiovascular (CV) death, restenosis, target lesion revascularization, and target vessel revascularization. Where available, we extracted both the unadjusted (crude) or minimally adjusted RR estimates and the maximally adjusted RR estimates. Where the RR was not reported we calculated crude RRs based on the number of events and total N within the HIV and the control groups, assuming a complete and comparable follow-up between the groups. The standard errors (SE) of the RRs were calculated using the following formula:

$$\sqrt{\frac{(n_1-x_1)/x_1}{n_1}+\frac{(n_2-x_2)/x_2}{n_2}}$$

where n1 = n HIV patients; X1 = n of HIV patients with the outcome (cases); n2 = n control patients; X2 = n of control patients with the outcome (cases).

We performed a random-effects meta-analysis to pool the RR estimates across the studies. We used the “metan” command developed by Michael J Bradburn, Jonathan J Deeks and Douglas G Altman (Centre for Statistics in Medicine, University of Oxford, Wolfson College Annexe, Linton Road, Oxford, UK) and implemented in Stata v.15. The command estimates between-study heterogeneity (tau2) using the DerSimonian-Laird method.[1] We performed sensitivity analyses by estimating between-study heterogeneity (tau2) using residual maximum likelihood (REML) method and calculated the variances (p-values and confidence intervals) of the pooled RR estimates applying modifications proposed by Knapp and Hartung.[2,3] For this sensitivity analysis, we used the “metareg” command in Stata developed by Roger B Harbord (Department of Social Medicine, University of Bristol, UK),[4] applying the default specifications for method of estimating the between-study heterogeneity (tau2) and calculating confidence intervals and p-values. REML is the default method for estimating tau2 in the “metareg” command. The “metareg” command by default applies modifications to the variance of the estimated pooled coefficients suggested by Knapp and Hartung (2003), accompanied by the use of Student *t* distribution in place of the standard normal distribution when calculating p-values and confidence intervals. These sensitivity analyses yielded comparable results. We considered odds ratios, risk ratios and hazard ratios as equivalent measure of RR. While odds ratios may not be good approximations to risk ratios for common outcomes, in this meta-analysis only two studies reported odds ratios, and the outcomes that the studies reported on were considered sufficiently rare.

The Stata code used to run the “metan” command along with an illustrative example of the corresponding Stata output for meta-analysis of all-cause mortality outcome is shown below:

metan log-HR SE-log-HR, eform randomi

© 2024 Haji M et al. *JAMA Network Open*.

All-cause mortality (RR HIV vs. Control)

Study	ES	[95% Conf.	Interval]	% Weight
Ren_2009	0.620	0.092	4.183	1.24
Cua_2014	1.690	1.074	2.660	12.07
Badr_2015	1.420	0.630	3.200	5.61
Carballo_2015	4.420	1.732	11.281	4.46
Marcus_2019	2.520	1.807	3.514	15.90
Shitole_2020	1.840	0.570	5.945	3.04
Postigo_2020	2.000	1.229	3.256	11.17
Parks_2021	1.320	1.215	1.434	24.33
Parikh_2023	1.210	1.031	1.421	22.18
D+L pooled ES	1.642	1.320	2.043	100.00

```
Heterogeneity chi-squared =  26.02 (d.f. = 8) p = 0.001
I-squared (variation in ES attributable to heterogeneity) =  69.3%
Estimate of between-study variance Tau-squared =  0.0492

Test of ES=1 : z=   4.46 p = 0.000
```

The Stata code used to run the "metareg" command along with an illustrative example of the corresponding Stata output for meta-analysis of all-cause mortality outcome is shown below:

xi: metareg log-HR, wsse(SE-log-HR) eform

All-cause mortality (RR HIV vs. Control)

```
Meta-regression                                        Number of obs   =        9
REML estimate of between-study variance                tau2            =   .08093
% residual variation due to heterogeneity              I-squared_res   =   69.25%
With Knapp-Hartung modification
```

lnhr	exp(b)	Std. Err.	t	P>|t|	[95% Conf.	Interval]
_cons	1.680019	.2215645	3.93	0.004	1.23947	2.277152

An example Stata command used to make the figures presented in the study is shown below:

```
metan log-HR SE-log-HR, eform ///
    title("RR of death after ACS/PCI HIV+ vs HIV-", ring(2) position(11) size(*0.6)) ///
    lcols(study N_HIV N_CON) randomi effect(RR) ///
    graphregion(color(white)) ///
    boxopt(msymbol(s) mcolor(black)) boxsca(75) ///
    pointopt(mcolor(none)) astext(50) textsize(125) ///
    ciopt(lwidth(thin)) ///
    xlabel(0.06, 0.12,0.25, 0.5,1,2,4,8) force  ///
    xtitle("RR (95% CI) HIV+ vs. HIV-", size(vsmall))
```

© 2024 Haji M et al. *JAMA Network Open*.

For comparison of baseline characteristics of the participants, we pooled summary characteristics reported in studies (e.g., mean age, male%, black%, white%, % smoker, % diabetes) using the study sizes as analytical weights, to provide estimates of weighted average means or percentages. The deltas, standard errors, and p-values comparing summary study-level characteristics (means or prevalence pooled across the studies) between HIV positive and HIV negative participants were calculated from a linear regression model of each variable upon HIV status, weighted by N for each study (i.e., fixed-effects meta-regression). For this we used the “regress” command in Stata.

The Stata code used to run the “regress” command along with an illustrative example of the corresponding Stata output for analysis of mean age difference by HIV status across the studies is shown below:

Xi: regress Age i.HIV [aw=n]

```
      Source |       SS           df       MS      Number of obs   =        28
-------------+----------------------------------   F(1, 26)        =     21.77
       Model |  20.2760892         1  20.2760892   Prob > F        =    0.0001
    Residual |  24.2207364        26  .931566786   R-squared       =    0.4557
-------------+----------------------------------   Adj R-squared   =    0.4347
       Total |  44.4968256        27  1.64803058   Root MSE        =    .96518

------------------------------------------------------------------------------
      av_age |      Coef.   Std. Err.      t    P>|t|     [95% Conf. Interval]
-------------+----------------------------------------------------------------
         hiv |  -11.07981   2.374911    -4.67   0.000    -15.96151   -6.198113
       _cons |   67.68496   .1829449   369.97   0.000     67.30891    68.06101
------------------------------------------------------------------------------
```


References:

1. DerSimonian, R., and N. Laird. 1986. Meta analysis in clinical trials. Controlled Clinical Trials 7: 177-188.
2. Knapp, G., and J. Hartung. 2003. Improved tests for a random effects meta-regression with a single covariate. Statistics in Medicine 22: 2693-2710.
3. IntHout J, Ioannidis JPA, Borm GF (2014). The Hartung-Knapp-Sidik-Jonkman method for random effects meta-analysis is straightforward and considerably outperforms the standard DerSimonian-Laird method. BMC Medical Research Methodology 14:25.
4. Sharp, S. 1998. sbe23: Meta-analysis regression. Stata Technical Bulletin 42: 16-22. Reprinted in Stata Technical Bulletin Reprints, vol. 7, pp. 148-155. College Station, TX: Stata Press.

© 2024 Haji M et al. *JAMA Network Open*.
