# Supplementary material for: Clinical Outcomes After Acute Coronary Syndromes or Revascularization Among People Living With HIV: A Systematic Review and Meta-Analysis
Source: JAMA Netw Open. 2024 May 14;7(5):e2411159. doi: 10.1001/jamanetworkopen.2024.11159 (PMC11094563; doi:10.1001/jamanetworkopen.2024.11159)
Supplement: Supplement 2. — Data Sharing Statement [file jamanetwopen-e2411159-s002.pdf]

Data Sharing Statement

Haji. Clinical Outcomes After Acute Coronary Syndromes or Revascularization Among People Living With HIV: A Systematic Review and Meta-Analysis. *JAMA Netw Open*. 2024;7(5):e2411159. doi:10.1001/jamanetworkopen.2024.11159

Data

Data available: Yes
Data types: Data (not involving human participants), Other (please specify)
Additional Information: Aggregate data based on published studies.
How to access data: Analysis dataset will be available from authors upon reasonable request.
When available: With publication

Supporting Documents

Document types: None

Additional Information

Who can access the data: Anyone requesting the data
Types of analyses: For purpose of replication.
Mechanisms of data availability: Without investigator support.
